# Revealing Patient Dissatisfaction With Health Care Resource Allocation in Multiple Dimensions Using Large Language Models and the International Classification of Diseases 11th Revision: Aspect-Based Sentiment Analysis

**DOI:** 10.2196/66344

**Published:** 2025-03-17

**Authors:** Jiaxuan Li, Yunchu Yang, Chao Mao, Patrick Cheong-Iao Pang, Quanjing Zhu, Dejian Xu, Yapeng Wang

**Affiliations:** 1 Faculty of Applied Sciences Macao Polytechnic University Macao Macao; 2 Department of Laboratory Medicine West China Hospital Sichuan University Chengdu China

**Keywords:** ICD-11, International Classification of Diseases 11th Revision, disease classification, patient reviews, patient satisfaction, ChatGPT, Sustainable Development Goals, chain of thought, large language model

## Abstract

**Background:**

Accurately measuring the health care needs of patients with different diseases remains a public health challenge for health care management worldwide. There is a need for new computational methods to be able to assess the health care resources required by patients with different diseases to avoid wasting resources.

**Objective:**

This study aimed to assessing dissatisfaction with allocation of health care resources from the perspective of patients with different diseases that can help optimize resource allocation and better achieve several of the Sustainable Development Goals (SDGs), such as SDG 3 (“Good Health and Well-being”). Our goal was to show the effectiveness and practicality of large language models (LLMs) in assessing the distribution of health care resources.

**Methods:**

We used aspect-based sentiment analysis (ABSA), which can divide textual data into several aspects for sentiment analysis. In this study, we used Chat Generative Pretrained Transformer (ChatGPT) to perform ABSA of patient reviews based on 3 aspects (patient experience, physician skills and efficiency, and infrastructure and administration)00 in which we embedded chain-of-thought (CoT) prompting and compared the performance of Chinese and English LLMs on a Chinese dataset. Additionally, we used the *International Classification of Diseases 11th Revision* (ICD-11) application programming interface (API) to classify the sentiment analysis results into different disease categories.

**Results:**

We evaluated the performance of the models by comparing predicted sentiments (either positive or negative) with the labels judged by human evaluators in terms of the aforementioned 3 aspects. The results showed that ChatGPT 3.5 is superior in a combination of stability, expense, and runtime considerations compared to ChatGPT-4o and Qwen-7b. The weighted total precision of our method based on the ABSA of patient reviews was 0.907, while the average accuracy of all 3 sampling methods was 0.893. Both values suggested that the model was able to achieve our objective. Using our approach, we identified that dissatisfaction is highest for sex-related diseases and lowest for circulatory diseases and that the need for better infrastructure and administration is much higher for blood-related diseases than for other diseases in China.

**Conclusions:**

The results prove that our method with LLMs can use patient reviews and the ICD-11 classification to assess the health care needs of patients with different diseases, which can assist with resource allocation rationally.

## Introduction

With the imbalance in regional economic development, there are 2 extreme problems in the accurate assessment of medical resources. On the one hand, health care management cannot accurately measure patients’ health care needs [1]. On the other hand, the assessment of medical resources cannot be accurate for each clinical department in hospitals, which leads to wastage of health care resources [2]. Research has shown that the improvement of health care resources enhances patient satisfaction [3]. To promote the sustainable development of the world, the United Nations has been advocating 17 Sustainable Development Goals (SDGs) since 2015. SDG 3 (“Good Health and Well-being”) and SDG 10 (“Reducing Inequalities”) suggest that the realization of universal health care coverage and avoiding lack or wastage of health care resources are urgent tasks [4-6]. SDG 9 (“Industry, Innovation and Infrastructure”) is about maintaining scientific research and innovation with the sustainable development of health care infrastructure and resources [7]. Overall, it is crucial to guarantee equitable access to health care services for all [8]. In this context, it is particularly important to assess health care resources based on patient needs within specific clinical departments [9]. For this reason, in this study, we planned to analyze patient dissatisfaction through their reviews after consultations to understand a macro view of their health care needs.

Chat Generative Pretrained Transformer (ChatGPT), a representative model of large language models (LLMs), was introduced in November 2022 [10], and its responsiveness as well as performance make it a possibility for further scientific research [11]. Several studies have pointed out that LLMs show strong performance in medical [12], financial [13], and education [14] fields, especially in the medical field, where ChatGPT shows reliable accuracy in questioning dialogues [15], diagnosing illnesses [16], suggesting treatments [17], and predicting prognosis [18]. Therefore, we wanted introduce LLMs into this health care needs study because with the powerful capability of LLMs, we can accomplish an in-depth assessment of health care resources.

Traditional sentiment analysis can determine whether the sentiment of a text is negative or positive, but it cannot determine sentiment from multiple perspectives [19]. However, some studies have shown that using aspect-based sentiment analysis (ABSA) can determine the affective state of a text from multiple perspectives, which is more suitable for us to determine the different perspectives of a patient’s health care needs [20-22], and therefore, we adopted ABSA for this study [20]. In this work, we proposed ABSA prompts that include “patient experience,” “physician skills and efficiency,” and “infrastructure and administration.” ABSA can more accurately indicate which of the patients’ health care needs are not being met by refining the direction of patient dissatisfaction with health care services, coupled with modern macro analysis of big data, in order to assess overall health care resources. However, in specific clinical application scenarios, different diseases have different disease characteristics, as well as their own different functional managers [23,24], so it is clinically important to analyze data from patient reviews of different disease types.

The *International Classification of Diseases 11th Revision* (ICD-11) coding data have been widely used in many fields [25], such as health statistics, public health monitoring, health care performance evaluation, and reimbursement [26]. ICD-11 is the latest international health information standard, adopted at the 72nd World Health Assembly in 2019, and was implemented in 2022. ICD-11 is a modern, integrated classification and nomenclature system with a modern digital medical model with application programming interfaces (APIs). One of the critical reasons why the World Health Organization (WHO) wants countries to move to ICD-11 is that a standardized classification based on the latest medical and scientific knowledge provides the best access to valuable data for prevention, resource allocation, or evaluation [2]. Several studies have shown that ICD-11 can be integrated with electronic medical records, and WHO’s multiple tools and guidelines can reduce training and implementation costs. Therefore, it is critical to expand ICD-11 research and program implementation worldwide [27].

As such, using ICD-11 to classify patients with different diseases, together with our ABSA prompts, it is possible to find differences in the satisfaction and dissatisfaction related to health care resource allocation among patients with different diseases. However, since the classification and statistical standards of medical diseases vary from one health care institution to another, normalization using ICD-11 as the standard will allow comparison of the same disease types to assess the balance of medical resources, as well as a comparison of different disease types to evaluate the differences in health care needs. By standardizing the classification of diseases, it will be possible to not only collect as much valid data as possible under the same standard but also conduct comparative studies of health care resources more effectively. Some researchers have indicated that they tested the coding accuracy of ICD-11 in a large-scale pilot study in 59 public hospitals in China. The results indicated that ICD-11 is well in line with Chinese disease classification habits, with the accuracy of the total coding domains and the accuracy of the word stem codes reaching 82.9% and 92.2%, respectively [28].

In this study, we analyzed patient comments using a LLM and determined the health care medical needs of patients with different diseases using ICD-11. We also discussed the reasons for these results in the context of our findings and the potential applications in clinical settings.

## Methods

### Architecture of the Experiment

**Figure 1:**
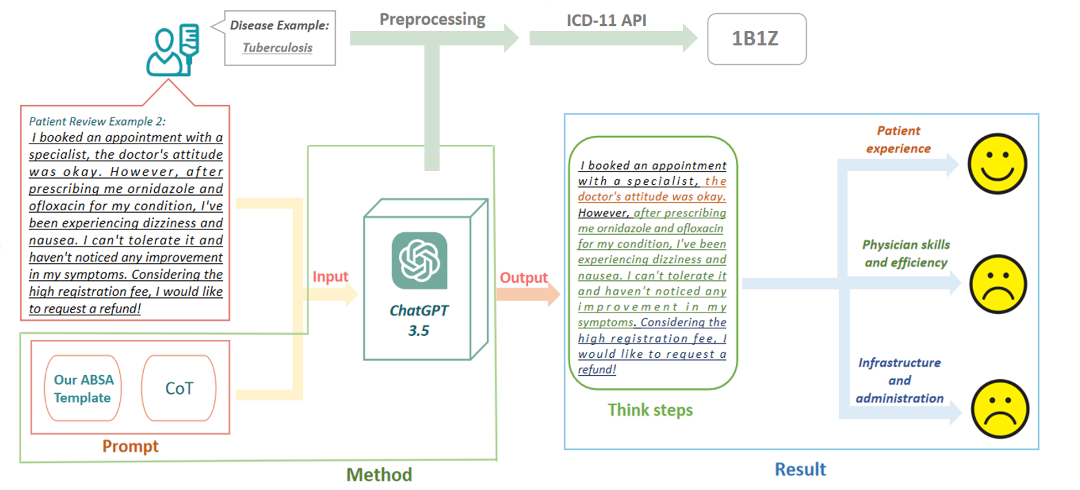
Proposed architecture for ABSA for analyzing patient data across different diseases using ChatGPT and the ICD-11 API. ABSA: aspect-based sentiment analysis; API: application programming interface; ChatGPT: Chat Generative Pretrained Transformer; CoT: chain of thought; ICD-11: *International Classification of Diseases 11th Revision*.

Figure 1 illustrates the proposed architecture for identifying medical needs from patient comments. In this architecture, we used our ABSA prompts in conjunction with chain-of-thought (CoT) prompting to generate questions fed into an LLM to analyze the content of patient comments. Considering the practicality and cost, we chose ChatGPT 3.5 Turbo as the LLM for our experiments as it is the most cost-effective choice. In the output, the model should be able to provide 3 categories of sentiment results. For example, the sample review presented in Figure 1 shows a positive sentiment for the category “patient experience” but a negative sentiment for the categories “physician skills and efficiency” and “infrastructure and administration.” Finally, we preprocessed the patients’ disease names through ChatGPT and typed the disease codes through the ICD-11 API. For example, the disease code 1B1Z returned from the API suggested that the disease belongs to Chapter 01 (the first digits of the code) of ICD-11.

### Data

The data used in this study came from Haodf Online [29], which is the most well-known online health care website in mainland China, with more than 6.1 million patients having uploaded their visit experiences [30]. Other studies have pointed out the data from online health care websites as important and valuable for research [31,32]. In addition, a study has shown that ChatGPT can directly process Chinese data without additional translation [33]. The comments contain the name of the disease, the type of treatment, the treatment outcome, the date of the visit, the location, the hospital name, the physician’s name, and patient comments. The datasets are in Chinese. The platform validates the comments to ensure that actual patients publish them.

We analyzed 552,764 comments on Haodf Online from November 2022 to June 2023. The data were not manually screened or deleted and spanned from October 2003 to June 2023. A total of 504,198 comments (91.2%) were retained after initial cleaning, including removing incomplete and duplicate data.

### Aspect-Based Sentiment Analysis Prompts

**Figure 2:**
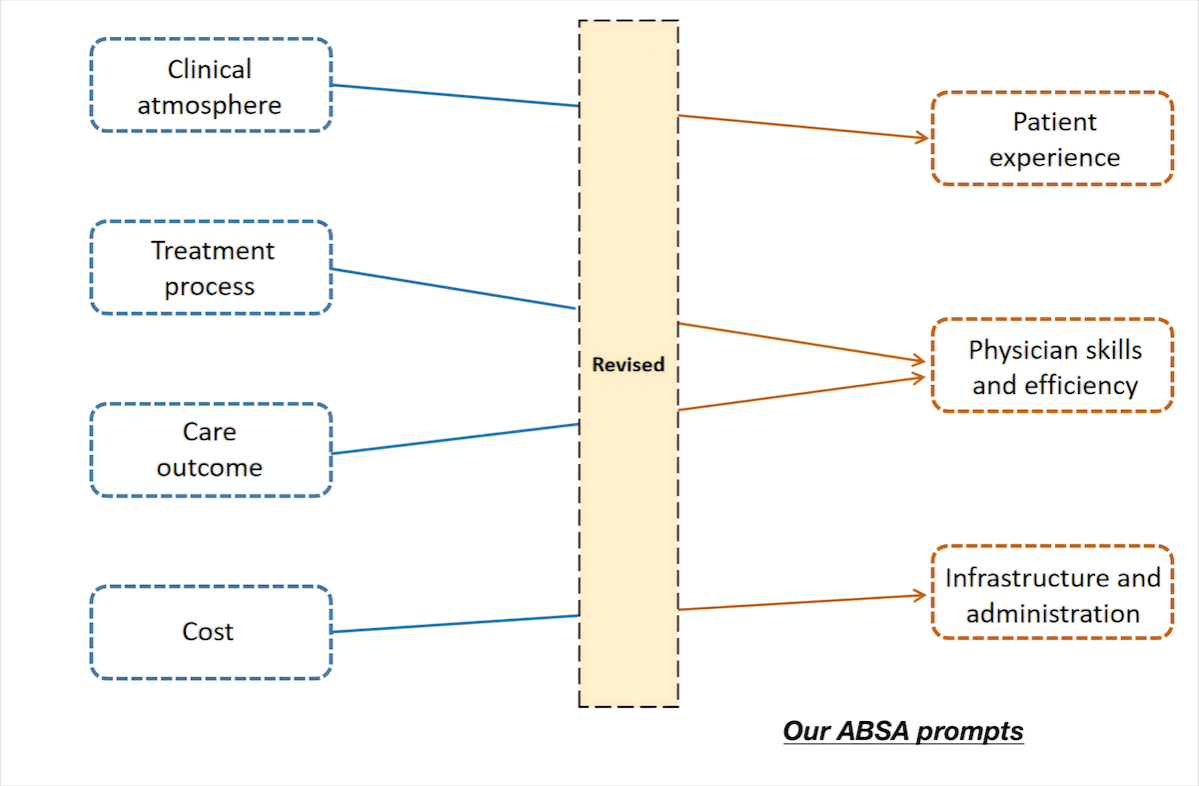
Illustration of formulating ABSA prompts from the dimensions of patient satisfaction. ABSA: aspect-based sentiment analysis.

Traditional sentiment analysis can only categorize sentiments for overall reviews and can only be performed with the entire content. However, positive and negative emotions may exist in long comments addressing different aspects of the medical process. Based on the existing literature and data, we designed prompts to address this issue [29,34]. Afrashtehfar et al [34] constructed an instrument that successfully assessed patient satisfaction in several ways. We optimized the latest version of their tool. Specifically, as shown in Figure 2, author QZ summarized 3 categories based on Afrashtehfar et al’s [34] findings and his own clinical experience and observations.

Specifically, “patient experience” includes perceptions of physicians, such as attitudes toward attending the hospital and the overall experience of attending the hospital. Second, “physician skill and efficiency” includes the physician’s treatment plan for the patient’s disease, the remission of the disease, and the physician’s treatment efficiency. Third, “infrastructure and administration” includes medical facility conditions, costs, the environment, hygiene, registration, and other administrative issues of the hospital. In summary, our ABSA prompts consisted of 3 categories (patient experience, physician skills and efficiency, and infrastructure and administration), all of which are clinically relevant and clinically meaningful.

### Chain of Thought

Some studies have shown that the use of CoT can alleviate problems such as hallucinations in an LLM to improve its accuracy [35-38]. We used the CoT technique to differentiate between the emotions related to the various topics in the comments. This process required logical reasoning to guide the LLM step by step through the thinking process, ensuring that steps were not skipped or omitted. With CoT, the LLM was guided to analyze the comments, confirm that each topic was been evaluated, and complete all the necessary steps in generating the final answer. Compared to outputs that do not use CoT, our approach can improve accuracy and facilitate checking the reasoning logic of the model.

**Figure 3:**
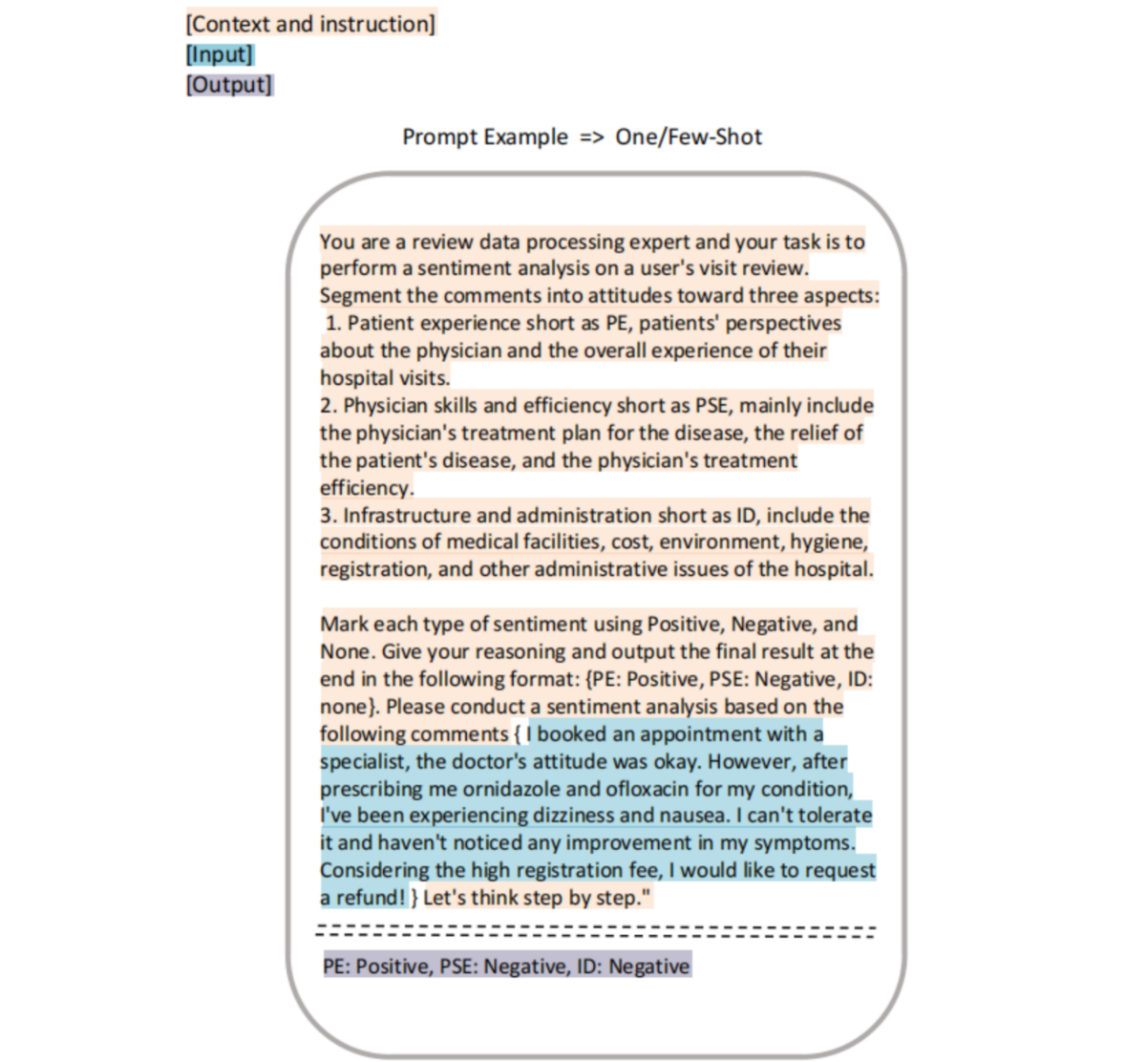
Prompt and CoT examples used in our work. CoT: chain of thought.

In addition, as shown in Figure 3, we used a zero-shot technique and added an example to the prompt to allow the model to learn to control its output. Once the LLM received our task, the CoT forced it to complete it step by step. Specifically, when ChatGPT received a patient’s comment, the model first extracted all the sentences related to “patient experience” from the comment and then analyzed the sentiment. After completing the sentiment analysis for the first category, the model extracted all sentences about “physician skills and efficiency” and performed sentiment analysis. Finally, the model extracted sentences about “infrastructure and administration” for sentiment analysis and output the results of all sentiment analyses. Our prompts were written in English, which aligns with the recommendations of other studies that suggest that using English in interactions with ChatGPT results in better performance. Other studies have noted that CoT performs better in English.

### ICD-11 API

It has been pointed out that the study of individual diseases is of little significance but that quantifying the distribution and determinants of different kinds of diseases is of significant importance [39,40]. Because diseases of the same kind tend to be highly interdependent, and similar diseases often share similar pathogenesis, risk factors, etc, it is much more important to perform clustered disease analyses than individual disease analyses [39,40]. Therefore, we intended to explore more meaningful information about patients’ health care needs by categorizing dispersed information about individual diseases through the ICD-11 API. It is also for this reason that some countries are piloting the possibility of health care system access to the ICD-11 API [28,41-44].

The ICD API is an HTTP-based REST API that allows programmatic access to the ICD [45]. Before the advent of the ICD-11 API, the only way to obtain a disease code was to search for it one at a time. In the 4-digit disease code, the first digit is the disease chapter code. There are 24 chapters in total. In version 2024, the International Classification of Functioning, Disability, and Health (ICF) was added as the second linearization of the API [46]. All linearization endpoints can access the ICF [46]. WHO provides a set of open source ICD-11 classification tools called API [47], we used Python's Requests library to call the linearlization search method provided by the API remotely.

Although in this version (2023-01), the ICD-11 API claims to have the capability of workin with the Chinese language, in our actual test, we found that the accuracy rate is lower than that for English when the input language is Chinese. One of the reasons for this is that when inputting Chinese, there are more requirements for the text to match. For example, we found that the ICD-11 API cannot recognize the Chinese characters for “disease” or “symptom” if they are missing (similar to issues reported in other work [28,48]). However, when we translated the terms into English and entered them into the ICD-11 API, it accurately recognized them and provided a disease code regardless of whether the Chinese character was or was not missing. In summary, we used ChatGPT 3.5 to translate Chinese disease names to solve the problem of the Chinese version of the ICD-11 API being too stringent in its search scope. In this way, the ICD-11 API generated correct results (as shown in the validation before).

### Evaluation Method

To more fully assess accuracy of our method, we used 2 approaches. First, we tested the performance of our method using manually labeled test sets and calculated precision, recall, and *F_1_*-scores. Evaluating the results using manual random sampling enhances their interpretability and makes them more comprehensive and credible. Therefore, we used a dual criterion to evaluate the output fully. Finally, we evaluated the cost and run time to assess the pertinence of our method in practical applications.

#### Model Performance

We manually annotated patient comment data using the 3 categories defined in our ABSA prompts. We labeled each category as “positive” or “negative” within the same comment. If the comment did not refer to a particular category, it was labeled “none.” Multiple authors with medical backgrounds performed the labeling. Two annotators worked independently (and one of them is a PhD candidate in the artificial intelligence [AI] field, while the other is a specialized physician at West China Hospital of Sichuan University). If the annotation results of the two annotators were inconsistent, we invited a professional doctor from a third-party hospital to resolve the conflict. We selected a test set of 2000 comments to assess precision, recall, and *F*_1_-scores. The *F*_1_-score combines precision and recall and is a widely used assessment metric in multicategory and uneven classification situations. Therefore, we use the *F*_1_-score to evaluate sentiment categorization methods. Its calculation is as follows:



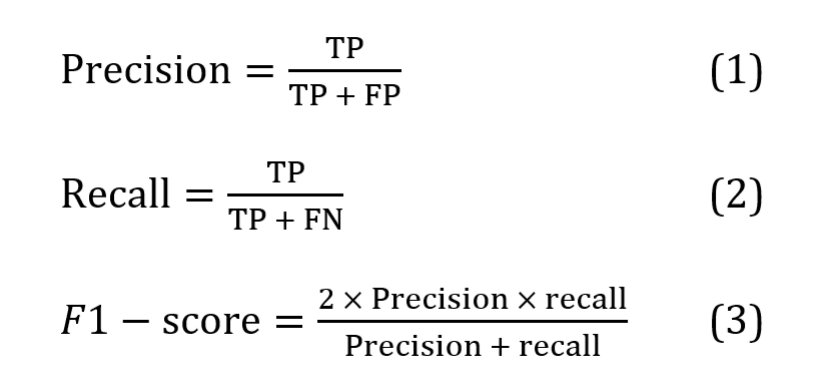



TP (true positive) stands for actual cases, indicating the number of samples that the model correctly predicted as positive cases; FN stands for false-negative cases, indicating the number of samples that the model failed to predict as positive cases correctly; and FP stands for false-positive cases, indicating the number of samples that the model incorrectly predicted as positive cases. To avoid sample imbalance affecting the accuracy of the conclusions, we chose to use the modified weighted *F*_1_-scores to calculate the model’s scores using Equation 4, where i is the index of the category, which is used to indicate the category of indicators currently being calculated, and w^i^ is the weight of category i, defined as the proportion of the sample size of that category to the total sample size.



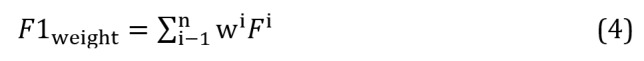



#### Reliability and Stability Evaluation

Although we used a manually labeled test set to assess the model’s accuracy, several studies have pointed to the instability and unreliability of LLMs. Therefore, we used a mixture of 3 sampling methods, whose merits have been pointed out by multiple studies, to assess the reliability and stability of the LLM model in our study. Specifically, mode 1 was simple random sampling, where 2000 pieces of data were drawn at a time within the total sample; mode 2 was incremental random sampling, where a total of 4 samples were taken, with 200, 400, 600, and 800 pieces of data, from the total sample; and mode 3 was isometric sampling, with a total of 4 draws each with 500 pieces of data taken from the total sample.

### Hyperparameterization

There are several adjustable hyperparameters in the GPT model we used, such as temperature, top_p, frequency_penalty, and presence_penalty. We did not change these hyperparameters but followed the default settings to ensure our experiments were broadly applicable. Thus, temperature was set to 1.0, top_p was set to 1.0, and frequency_penalty and presence_penalty were set to 0.

### Ethical Considerations

The dataset in this study was anonymized, and ethical clearance was obtained from the Macao Polytechnic University (approval no: HEA005-FCA-2024).

## Results

### Model Comparisons

Results showed that the accuracy of GPT-4o is slightly higher than that of ChatGPT 3.5, while its running time and costs are substantially higher than those of ChatGPT 3.5, which is not consistent with the research goal of sustainable development. GPT-4o took 104 minutes to process 1000 patient reviews, while ChatGPT 3.5 took only 71 minutes. In addition, GPT-4o cost US $1.7 to run 1000 patient reviews, while ChatGPT 3.5 cost only US $0.8 per 1000 reviews. The open source model Qwen-7B cost US $0 in comparison. In addition, the accuracy of GPT-4o was only 0.004 times higher than that of ChatGPT 3.5, while the accuracy of Qwen-7B was too low at 0.682 to meet the research criteria of this study. Therefore, we selected ChatGPT 3.5 as the experimental base model.

**Table 1:** Accuracy, run time, and cost of the 3 candidate models.

Model	Accuracy	Run time^a^ (minutes)	Cost^a^ (US $)
ChatGPT^b^ 3.5	0.907	71	0.8
GPT-4o	0.911	104	1.7
Qwen-7B	0.682	73	0

^a^Per 1000 reviews.

^b^ChatGPT: Chat Generative Pretrained Transformer.

### Model Performance

We tested the model on a test set of 2000 patient reviews with labels. To demonstrate the variability of the study, we introduced 95% CIs. As shown in Table 2, the model’s weighted total results indicated a precision of 0.907 (95% CI 0.865-0.949), an *F*_1_-score of 0.793 (95% CI 0.784-0.802), and a recall of 0.748 (95% CI 0.737-0.759). Thus, the model fulfilled the quality requirements of our experiments.

**Table 2:** Results for model precision, *F*_1_-score, and recall by category.

Category	*F*_1_-score	Precision	Recall
Patient experience	0.965	0.971	0.970
Physician skills and efficiency	0.899	0.948	0.879
Infrastructure and administration	0.405	0.860	0.394
Weighted total	0.793	0.907	0.748

### Model Reliability and Stability

We used 3 different manual test sampling methods in this work. As shown in Table 3, the average accuracies of the 3 different sampling methods were 90.1%, 88.5%, and 89.4%, respectively. This was close to our accuracy of 0.907 described before, again confirming the reliability of both evaluation methodologies in the experiment.

**Table 3:** Model accuracy results for 3 manual random sampling methods.

Mode and characteristics		Sampling			
		First	Second	Third	Fourth
**Mode 1 (entire sample accuracy: 90.1%)**					
	Sample size, n (%)	2000 (100)	—^a^	—	—
	Patient experience accuracy (%)	92.2	—	—	—
	Physician skills and efficiency accuracy (%)	89.4	—	—	—
	Infrastructure and administration accuracy (%)	88.6	—	—	—
**Mode 2 (entire sample accuracy: 88.5%)**					
	Sample size, n (%)	200 (10)	400 (20)	600 (30)	800 (40)
	Patient experience accuracy (%)	94.0	90.3	92.5	91.3
	Physician skills and efficiency accuracy (%)	90.5	88.8	89.8	88.6
	Infrastructure and administration accuracy (%)	83.0	82.8	86.2	84.3
**Mode 3 (entire sample accuracy: 89.4%)**					
	Sample size, n (%)	500 (25)	500 (25)	500 (25)	500 (25)
	Patient experience accuracy (%)	93.8	95.8	89.6	90.8
	Physician skills and efficiency accuracy (%)	88.8	92.4	95.4	90.4
	Infrastructure and administration accuracy (%)	83.2	84.4	81.8	86.0

^a^Not applicable.

### ICD-11 API Accuracy

We used Python to randomly extract 1000 disease names with ICD-11 codes from the datasets with ICD-11 chapter labels. Under the guidance of one of the authors of this paper, from the West China Hospital of Sichuan University, these data were reviewed for accuracy, and the results showed that 954 (95.4%) were correct, while 46 (4.6%) were incorrect; this accuracy of 95.4% met our experimental requirements.

### Chapters of ICD-11

**Figure 4:**
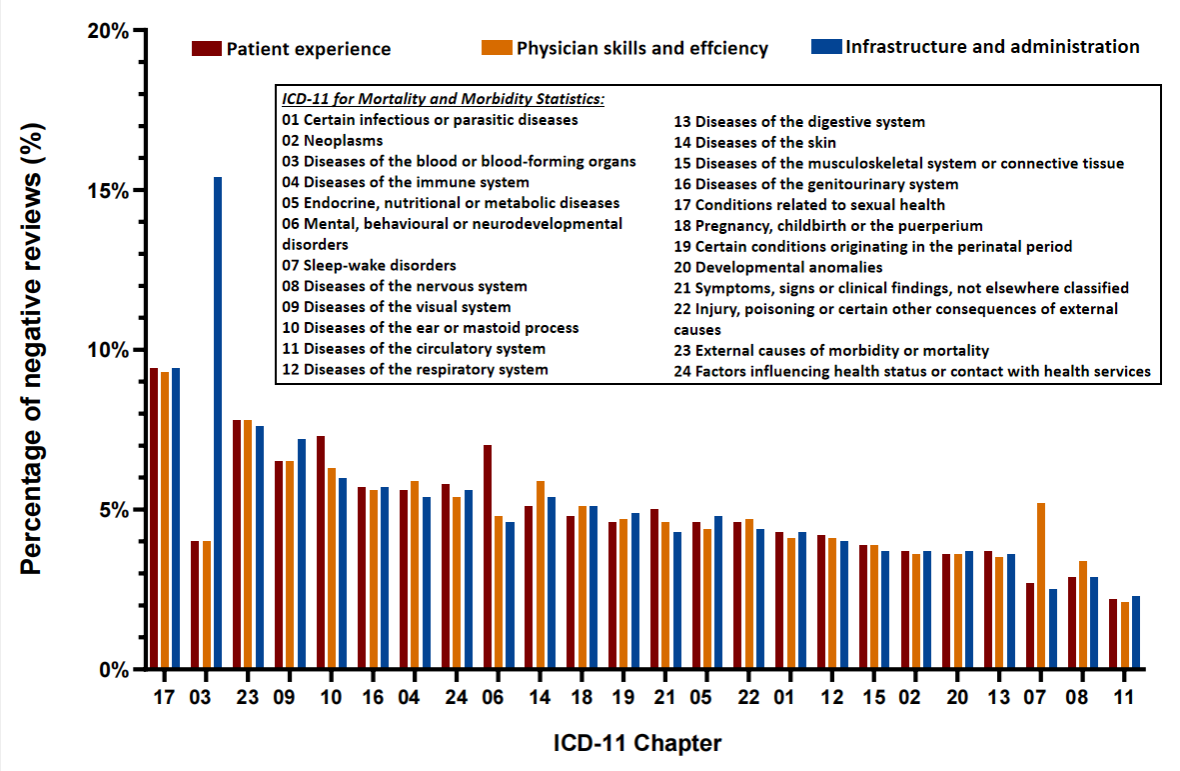
Percentage of negative comments by chapter in ICD-11. The x-axis was sorted by the average percentage of negative reviews on all aspects. ICD-11: *International Classification of Diseases 11th Revision*.

We calculated the distribution of the different types of diseases in ICD-11 across the 3 different categories of health care needs in our ABSA prompts. As shown in Figure 4, the diseases in ICD-11 Chapter 3 (“Diseases of the Blood or Blood-Forming Organs”) showed a high degree of variability, with the percentage of negative comments on the “infrastructure and administration” category exceeding 15%, which is significantly higher than in all other chapters. However, the percentage of negative comments on “patient experience” and “physician skills and efficiency” was only about 4%, which was only higher than for diseases of the nervous system, sleep-wake disorders, and diseases of the circulatory system (ICD-11 Chapters 8, 7, and 11, respectively).

Next, to explore the overall patient demand by disease, we calculated the average percentage of negative ABSA comments and ranked them in descending order. As shown in Table 4, most of the chapters had similar rates of poor ratings for all 3 categories of health care needs in our ABSA prompts. In chapters on diseases of the blood or blood-forming organs; mental, behavioural, or neurodevelopmental disorders; and sleep-wake disorders (Chapters 3, 6, and 7, respectively), there was a significant difference in a single category. In the chapter on mental, behavioural, or neurodevelopmental disorders (Chapter 6), the “patient experience” category had a negative review percentage of about 7%, while the remaining 2 categories were below 5%. In the chapter on sleep-wake disorders (Chapter 7), the percentage of negative comments for “physician skills and efficiency” exceeded 5%, while the remaining 2 categories were below 3%.

**Table 4:** Percentage of negative comments by patients with different diseases based on ICD-11^a^ chapters.

ICD-11 chapter	Patient experience (%)	Physician skills and efficiency (%)	Infrastructure and administration (%)	Average (%)
17	9.40	9.30	9.40	9.37
3	4.00	4.00	15.40	7.80
23	7.80	7.80	7.60	7.73
9	6.50	6.50	7.20	6.73
10	7.30	6.30	6.00	6.53
16	5.70	5.60	5.70	5.67
4	5.60	5.90	5.40	5.63
24	5.80	5.40	5.60	5.60
6	7.00	4.80	4.60	5.47
14	5.10	5.90	5.40	5.47
18	4.80	5.10	5.10	5.00
19	4.60	4.70	4.90	4.73
21	5.00	4.60	4.30	4.63
5	4.60	4.40	4.80	4.60
22	4.60	4.70	4.40	4.57
1	4.30	4.10	4.30	4.23
12	4.20	4.10	4.00	4.10
15	3.90	3.90	3.70	3.83
2	3.70	3.60	3.70	3.67
20	3.60	3.60	3.70	3.63
13	3.70	3.50	3.60	3.60
7	2.70	5.20	2.50	3.47
8	2.90	3.40	2.90	3.07
11	2.20	2.10	2.30	2.20

^a^ICD-11: *International Classification of Diseases 11th Revision*.

As also listed in Table 4, chapters on diseases of the blood or blood-forming organs (Chapter 3), conditions related to sexual health (Chapter 17), external causes of morbidity or mortality (Chapter 23), and diseases of the visual system (Chapter 9) had the top 4 highest percentages of negative comments, exceeding 9.8%, 7.8%, 7.7%, and 6.7%, respectively. The direction of “infrastructure and administration” in diseases of the visual system (Chapter 9) and “patient experience” in diseases of the ear or mastoid process (Chapter 10) reached 7.2% and 7.3%, respectively, significantly higher than the other 2 categories. In addition, chapters on diseases of the digestive system, diseases of the nervous system, sleep-wake disorders, and diseases of the circulatory system (Chapters 13, 8, 7, and 11, respectively) had the lowest poor ratings, which were below 4%, 3.5%, 3%, and 2.5%, respectively, except for the classification of “physician skills and efficiency” in sleep-wake disorders (Chapter 7).

**Figure 5:**
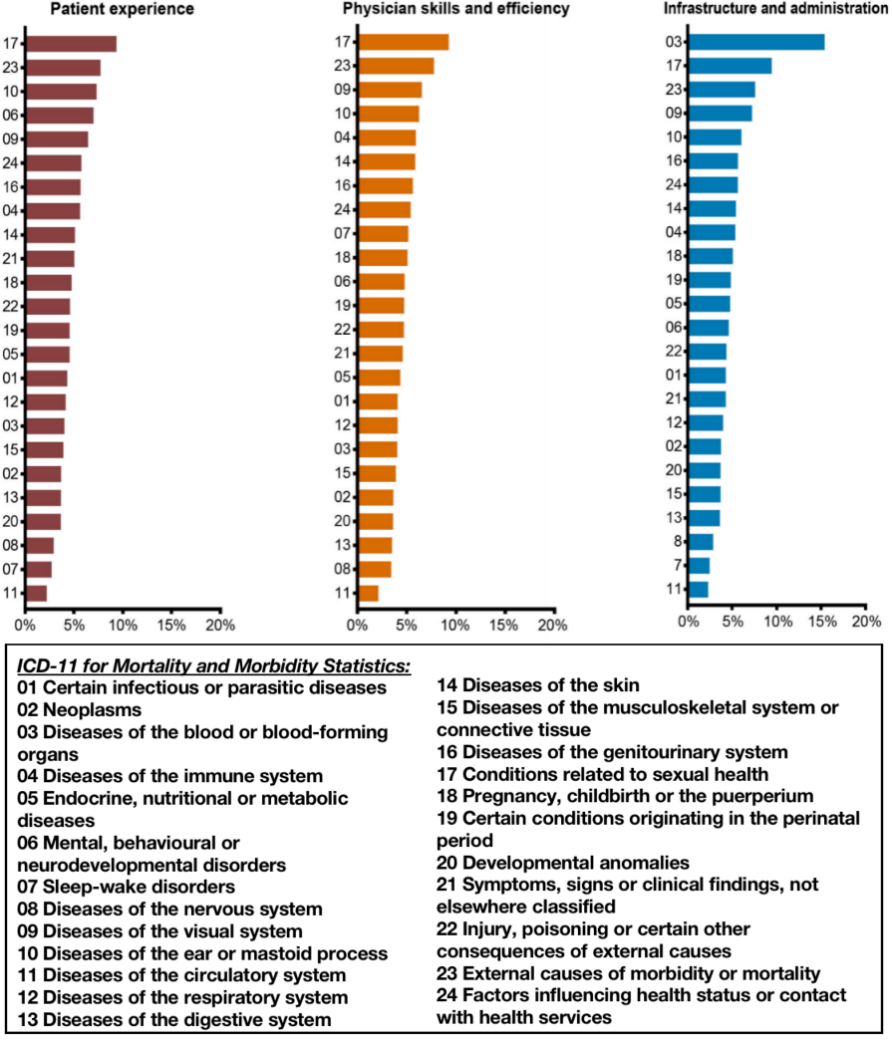
Percentage of negative reviews by ICD-11 chapter. ICD-11: *International Classification of Diseases 11th Revision*.

Figure 5 shows a better visualization of the percentage of negative reviews in each category in the ABSA prompts and the differences between the different chapters. In the “patient experience” category, the 3 chapters on conditions related to sexual health, external causes of morbidity or mortality, and diseases of the ear or mastoid process (Chapters 17, 23, and 10, respectively) had the highest percentage of negative comments, which exceeded 9%, 7.5%, and 7%, respectively. In addition, chapters on mental, behavioural, or neurodevelopmental disorders; diseases of the visual system; factors influencing the health status or contact with health services; diseases of the genitourinary system; diseases of the immune system; and diseases of the skin and symptoms not elsewhere classified (Chapters 6, 9, 24, 16, 4, 14, and 21, respectively) had negative comments exceeding 5 %, for a total of 9 chapters. Except for these 9 chapters, the rest were below 5%, and the 3 chapters with the lowest percentage of negative comments were on diseases of the nervous system, sleep-wake disorders, and diseases of the circulatory system (Chapters 8, 7, and 11, respectively), at less than 3%, 3%, and 2.5%, respectively.

In the “physician skills and efficiency” category, chapters on conditions related to sexual health, external causes of morbidity or mortality, and diseases of the visual system (Chapters 17, 23, and 9, respectively) had the highest percentage of negative comments at 9%, 7.5%, and 6.5%, respectively. In addition, chapters on diseases of the ear or mastoid process , diseases of the immune system, diseases of the skin, diseases of the genitourinary system, factors influencing the health status or contact with health services, sleep-wake disorders, and diseases of the nervous system (Chapters 10, 4, 14, 16, 24, 7, and 8, respectively) had more than 5% of negative comments, for a total of 10 chapters. In addition, the 3 chapters with the lowest negative comment percentage were on diseases of the digestive system, diseases of the nervous system and diseases of the circulatory system (Chapters 13, 8, and 11, respectively) at about 3.5%, 3.5%, and 2%, respectively. The rest of the chapters had a negative comment percentage between 5 and 3.5%.

Finally, in the “infrastructure and administration” category, the 3 chapters with the highest percentage of negative comments were on diseases of the blood or blood-forming organs, conditions related to sexual health, and external causes of morbidity or mortality (Chapters 3, 17, and 23, respectively), at 15%, 9%, and 7.5%, respectively. Additionally, 15% was the highest percentage of negative comments among all 3 categoriess. The next highest percentage of negative comments was found for chapters on diseases of the visual system, diseases of the ear or mastoid process, diseases of the genitourinary system, factors influencing the health status or contact with health services, diseases of the skin, diseases of the immune system and pregnancy, childbirth or the puerperium (Chapters 9, 10, 16, 24, 14, 4, and 18, respectively), with the following highest percentage of negative comments being found for chapters on diseases of the circulatory system, sleep-wake disorders, and diseases of the nervous system (Chapters 11, 7, and 8, respectively), at less than 3%.

**Figure 6:**
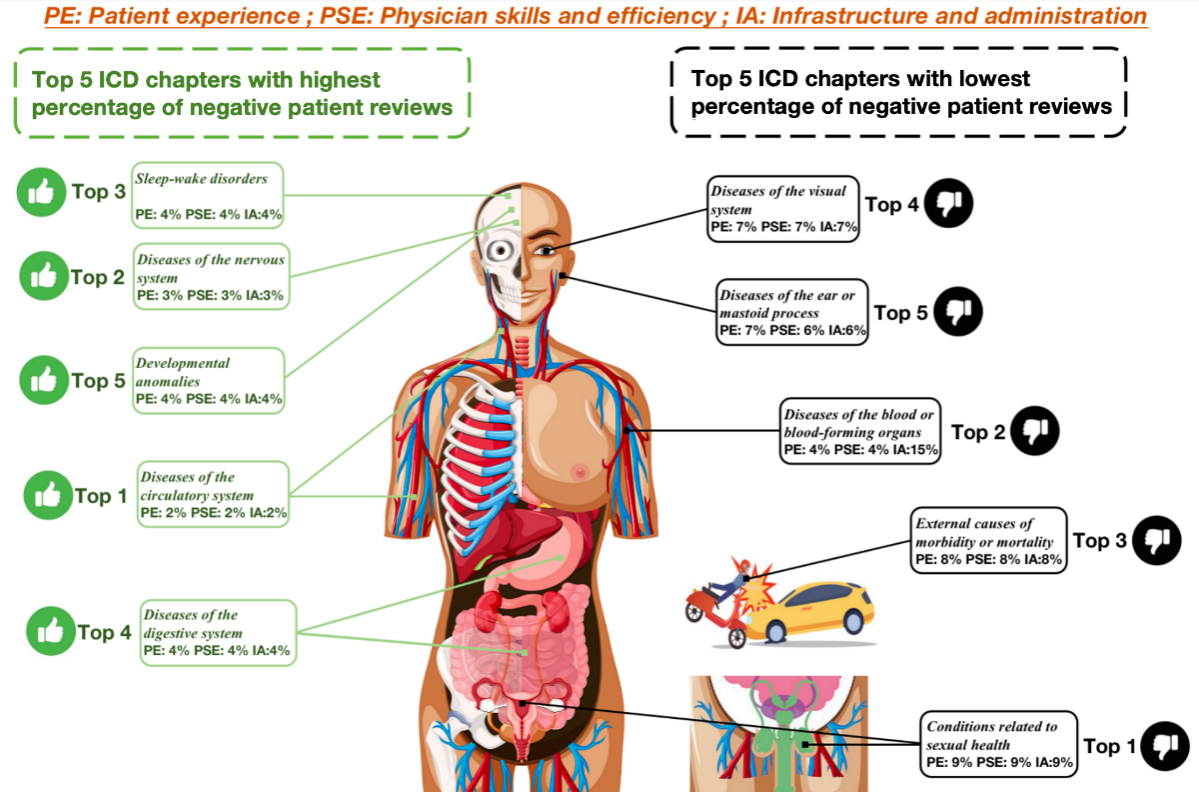
Visualization of parts of the body systems with the highest and lowest percentages of negative patient reviews by ICD-11 chapter. ICD-11: *International Classification of Diseases 11th Revision*.

To visually observe the relationship of ABSA results among diseases, we visualized the top 5 ICD-11 chapters with the highest and the lowest percentage of negative patient reviews using a body map. Based on the visualization, we obtained a clearer image of different body parts with the highest and lowest percentages of negative reviews. As shown in Figure 6, most of the top 5 chapters with the lowest percentages focus on diseases near the brain, including neurological disorders, sleep disorders, and developmental disorders.

## Discussion

### Principal Findings

#### Model Accuracy and Feasibility

The results showed that our model achieves an *F*_1_-score, precision, and recall of 0.793, 0.907, and 0.748, respectively, proving the effectiveness of our combination of ABSA prompts and CoT architecture. In addition, the model achieved 90.1%, 88.5%, and 89.4% accuracy in the stability and reliability tests conducted in random sampling modes 1, 2, and 3, respectively. We used an earlier version of LLMs (ChatGPT-3.5-turbo-0613), which was released in 2023. This proved that the initial version of GPT was already sufficient for our task, and it was expected that newer versions of the model can also be used for this type of task.

#### Differences in Patients’ Health Care Needs Across ICD-11 Chapters

Overall, across all classifications, patients with sexual health–related illnesses had high health care needs and showed high levels of dissatisfaction with all aspects. Based on the clinical setting and research in China [49-51], we hypothesize that this may be related to the extremely private nature of the disease, which leads to shame and low self-esteem, which, in turn, leads to a sensitive and vulnerable mindset and, therefore, extreme concern for the physician’s attitude, skills, and the environment in which the consultation takes place. In contrast, patients with circulatory disorders expressed satisfaction with current medical resources, suggesting that China has an advantage in researching and treating this type of disease.

Second, although the overall medical needs of patients with sleep-wake disorders are not high, patients with these disorders show a higher demand for physician skills and efficiency, probably because it is difficult to see immediate results in the treatment of these patients and because insomnia can lead to neurasthenia, etc, which can have a significant impact on work and life. Therefore, there is a strong demand for efficacy for such types of conditions.

In addition to the aforementioned diseases, patients with ear or mastoid disorders also have a relatively high demand for patient experience. This may be because ear diseases make patients wary and uncomfortable with all the sounds in their environment and, therefore, extremely sensitive to the attitude and tone of the physician’s dialogue. Furthermore, immune system disorders have a higher need for physician skill and efficiency, which may be related to the degree of difficulty in treating the diseases, as immune system disorders are generally more difficult to cure. Therefore, there is generally a robust and heartfelt expectation from patients regarding the physician’s skill.

Lastly, neurological or hematopoietic organ diseases lead the way regarding the “infrastructure and administration” category. This may be because these diseases depend highly on advanced hospital infrastructure, such as specific hemodialysis equipment. In short, each disease has different medical needs. Therefore, medical management can allocate or solve problems according to the medical needs of various diseases. If one needs to continue to explore the specific causes of a problem, as proposed in a previous work [52], one can do so downstream of the task, such as word frequency analysis and word cloud construction. In this way, we can uncover specific negative reasons in patient comments.

### Implications for Sustainable Development Goals

Our work on analyzing patient comments using ICD-11 and LLMs to identify health care needs for different diseases has important implications for the SDGs. First, in SDG 3 (“Good Health and Well-being”), the precise analysis of patient comments can provide a more accurate understanding of the specific needs of different diseases, promote accurate consultation and treatment in health care units, and improve the quality of their health care services. In SDG 9 (“Industry, Innovation and Infrastructure”), our research demonstrates the use of LLMs and ICD-11 for health care innovation to improve diagnosis and treatment efficiency, promote smart health care infrastructure, and enhance the accessibility and efficiency of health care services in various ICD-11 chapters. In addition, for SDG 10 (“Reducing Inequalities”), by revealing the health needs and health care disparities among different groups, we can help develop more targeted health policies to reduce health care inequalities and ensure that all people have access to appropriate health care, as well as emphasizing the importance of the patient’s voice and promoting inclusive health care.

Finally, for SDG 17 (“Partnerships for Goal Achievement”), this research highlights the importance of interdisciplinary collaboration, combining medicine, data science, and AI technologies to encourage multiple parties, including public health organizations, academic institutions, and technology companies, to work together to address health challenges worldwide.

In conclusion, our research not only has important application value in the medical field but also provides valuable insights into the achievement of the SDGs and promotes the innovation and development of health care services through the full use of modern scientific and technological tools to enhance health worldwide and promote the sustainable development of society.

### Potential Applications of the Assessment of Health Care Services

Several studies have shown that physicians or hospitals in the United States are incentivized by patient scores or ratings [53-55], which helps them provide efficient, high-quality care [56,57]. At the same time, these scores or ratings are made public on medical websites, and people can choose their preferred departments or hospitals through the ratings. In China, there is a need for ratings for hospitals [58], and although some medical websites have ratings for physicians or hospitals, the ratings on websites could be more sketchy and include only a small number of physicians or hospitals [59]. Therefore, governments and health authorities can use our approach to obtain fine-grained ratings of each health care unit to enhance monitoring and management, which would benefit both patients and the administration.

From the patients’ perspective, aggregating satisfaction and dissatisfaction scores based on ICD-11 codes can help them select health care providers based on their preferences. For example, if a patient is more concerned about the facilities and customer experience of a hospital, they can choose a hospital with a lower ratio of negative reviews in the “infrastructure and administration” category. In addition, as shown in Multimedia Appendix 1, the health care administration can obtain ABSA results with ICD-11 codes using our method and use it for continuous improvements. The result with ICD-11 codes can be shown as a dashboard, and management can identify areas with high satisfaction and dissatisfaction. Patient reviews in such areas can be further analyzed using natural language processing techniques (eg, word frequency analysis or topic modeling) to identify the root causes.

Taking our datasets as an example, assuming that the they originate from the same hospital, management can prioritize the verification and optimization of health care resources for equipment and environments in different departments with a high percentage of negative comments in the “infrastructure and administration”category (eg, ICD-11 Chapters 1, 17, and 23 in this case). Second, management can organize quality education and psychoeducation for doctors to optimize departments with a high percentage of negative comments in the “patient experience” category (eg, ICD-11 Chapters 10, 17, and 23). Finally, management can provide systematic training to doctors and organize professional meetings to address departments with a high percentage of negative comments in the “physician skills and efficiency” category (eg, ICD-11 Chapters 9, 17, and 23). These evaluation cycles can be iterated over time for checking whether there is any improvement.

### Limitations

This study has several limitations. Since this study proposed a new ABSA template, there are no other datasets with annotations to be compared and cross-validated with this study for now; therefore, it is not possible to validate whether this method can be applied to datasets in other languages. In addition, it is not possible to verify whether it is meaningful to apply the ABSA in this study to regions with different health care environments. In terms of application, we might be able to make in-depth analyses by comparing data from multiple regions in the future. Finally, to prove that the problem of SDGs has been solved requires a comprehensive evaluation framework and evaluation method [32,60]. In the future, we anticipate collaboration with multiple hospitals to conduct a more comprehensive evaluation and practice in a specific clinical setting to determine the positive impact of this study on health care resource allocation and SDGs.

### Conclusion

We analyzed the distribution of health care resources by disease through an LLM of patient reviews. In addition, we combined the results with the ICD-11 classification and found that conditions related to sexual health have the lowest overall patient satisfaction. In contrast, patient satisfaction is highest among patients with diseases of the circulatory system. Our approach demonstrates the possibility of using an LLM to understand patients’ needs and extract aspect-based information from reviews written by them and health consumers.

## Appendix

Multimedia Appendix 1 Two potential directions of the application of our method. ABSA: aspect-based sentiment analysis; ICD-11: *International Classification of Diseases 11th Revision*.

## Abbreviations

ABSA: aspect-based sentiment analysis
AI: artificial intelligence
API: application programming interface
ChatGPT: Chat Generative Pretrained Transformer
CoT: chain of thought
ICD-11: *International Classification of Diseases 11th Revision*
ICF: International Classification of Functioning, Disability, and Health
LLM: large language model
SDG: Sustainable Development Goal

## Data Availability

The datasets analyzed during this study are available from the corresponding author upon reasonable request.
