# Supplementary figures and images for: Revealing Patient Dissatisfaction With Health Care Resource Allocation in Multiple Dimensions Using Large Language Models and the International Classification of Diseases 11th Revision: Aspect-Based Sentiment Analysis
Source: J Med Internet Res. 2025 Mar 17;27:e66344. doi: 10.2196/66344 (PMC11959199; doi:10.2196/66344)

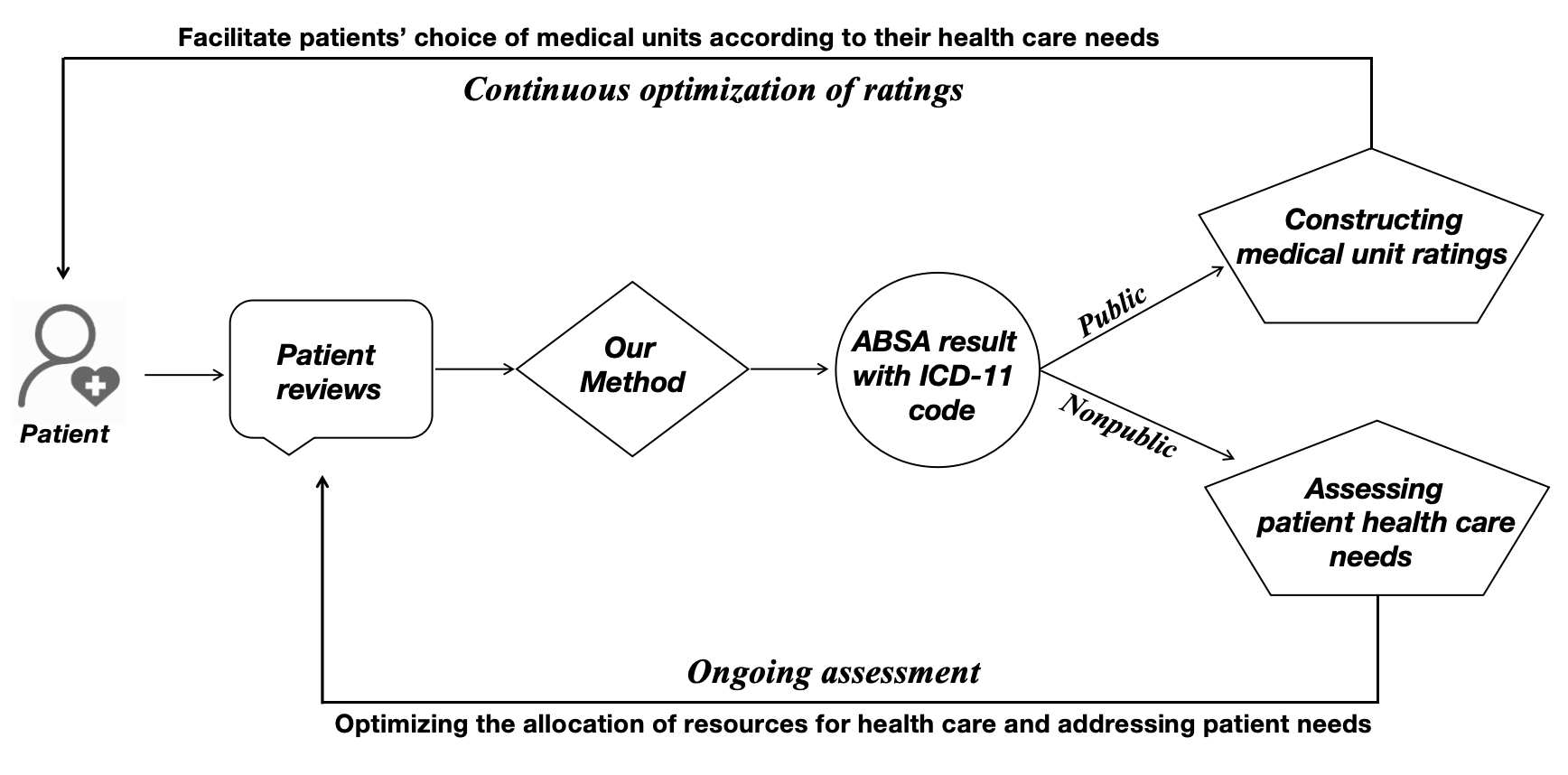

Supplement: Multimedia Appendix 1 [file jmir_v27i1e66344_app1.png]
